# Precision Medicine for Hypertension Patients with Type 2 Diabetes via Reinforcement Learning

**DOI:** 10.3390/jpm12010087

**Published:** 2022-01-11

**Authors:** Sang Ho Oh, Su Jin Lee, Jongyoul Park

**Affiliations:** 1 Research Center of Electrical and Information Technology, Seoul National University of Science and Technology, Seoul 01811, Korea; shoh0320@seoultech.ac.kr; 2 Department of Internal Medicine, Seoul Red Cross Hospital, Seoul 03181, Korea; kibalhae82@gmail.com; 3 Department of Applied Artificial Intelligence, Seoul National University of Science and Technology, Seoul 01811, Korea

**Keywords:** precision medicine, hypertension, diabetes, reinforcement learning, Q-learning, treatment recommendation, healthcare management

## Abstract

Precision medicine is a new approach to understanding health and disease based on patient-specific data such as medical diagnoses; clinical phenotype; biologic investigations such as laboratory studies and imaging; and environmental, demographic, and lifestyle factors. The importance of machine learning techniques in healthcare has expanded quickly in the last decade owing to the rising availability of vast multi-modality data and developed computational models and algorithms. Reinforcement learning is an appealing method for developing efficient policies in various healthcare areas where the decision-making process is typically defined by a long period or a sequential process. In our research, we leverage the power of reinforcement learning and electronic health records of South Koreans to dynamically recommend treatment prescriptions, which are personalized based on patient information of hypertension. Our proposed reinforcement learning-based treatment recommendation system decides whether to use mono, dual, or triple therapy according to the state of the hypertension patients. We evaluated the performance of our personalized treatment recommendation model by lowering the occurrence of hypertension-related complications and blood pressure levels of patients who followed our model’s recommendation. With our findings, we believe that our proposed hypertension treatment recommendation model could assist doctors in prescribing appropriate antihypertensive medications.

## 1. Introduction

Precision medicine is a new approach to understanding health and disease based on patient-specific data such as medical diagnoses, clinical phenotype, biologic investigations such as laboratory studies and imaging, and environmental, demographic, and lifestyle factors. These data are called multi-modal when combined since they provide information from various domains. The exponential increase in the amount of electronic health data that can now be collected for each patient, in large part due to the advent of new technologies in the fields of medicine, genetics, metabolic, and imaging, among others, has had a significant impact on the evolution of precision medicine [1]. The number and diversity of diagnostic tests generate an enormous amount of data that is difficult to comprehend and evaluate for a single patient and considerably more difficult to comprehend and analyze in a dataset including data from numerous patients. Fortunately, when more complex diagnostic tests were created, the discipline of machine learning evolved as well, providing for more efficient storage and analysis of these vast volumes of data than ever before. These two advancements work together, with machine learning approaches utilizing the enormous volumes of deep data produced in the healthcare system to promote precision medicine diagnostics and therapies [1].

The importance of machine learning techniques in healthcare has expanded quickly in the last decade, owing to the rising availability of vast multi-modality data and developed computational models and algorithms [2,3,4]. This new trend has sparked increased interest in using advanced data analytics and machine learning methodologies in a range of healthcare settings [5,6,7]. As a subset of machine learning, reinforcement learning has made significant theoretical and technical advances in generalization, representation, and efficiency in recent years [8]. It leads to an increase in its applicability to real-world problems such as gaming, robotics control, autonomous driving, computer vision, and biological data analysis [8,9,10,11].

Reinforcement learning is an appealing method for developing efficient policies in various healthcare areas where the decision-making process is typically defined by a long period or a sequential process [12]. In reinforcement learning problems, an agent takes action based on its present state at each time step, and the environment provides evaluative feedback and the new state. The agent aims to develop an optimal policy that maximizes the amount of money it earns over time. Reinforcement learning is particularly well adapted to systems with intrinsic temporal delays, such as those in which decisions must be made without immediate knowledge of their effectiveness. A medical or clinical treatment regime is typically made up of a series of decisions to determine the best course of action, such as treatment type, drug dosage, or re-examination timing, based on a patient’s current health status and prior treatment history, to maximize the patient’s long-term benefits. Unlike traditional randomized controlled trials, which derive treatment regimens from the average population response, reinforcement learning can be tailored to achieve precise treatment for individual patients with high heterogeneity in response to treatment due to differences in disease severity, personal characteristics, and drug sensitivity. Furthermore, reinforcement learning can develop optimal policies based solely on prior experiences, with no prior understanding of the mathematical model of biological systems required. This makes reinforcement learning more practical than other existing machine learning approaches in healthcare domains. Building an accurate model for the human health system and the responses to administered treatments can be difficult, if not impossible, due to nonlinear, varying, and delayed interactions between treatments and human bodies [8].

Hypertension become one of the major cause of death and disability-adjusted life-years worldwide, with more cardiovascular deaths than other modifiable risk factors [13]. People with hypertension are more likely to have comorbid chronic illnesses. Because the requirement to address concomitant chronic illnesses in addition to patients’ hypertension-specific treatment goals poses a significant obstacle for efficient hypertension management, type 2 diabetes (T2DM) is the most prevalent multi-morbidity for hypertension patients [14]. With this fact, we focused on the hypertension patients with T2DM to deal with severe state patients. Despite the availability of various medications, hypertension is poorly controlled, with large gaps in hypertension knowledge, antihypertensive therapy adoption, and blood pressure control adequacy [15,16]. Recent papers and treatment guidelines on precision medicine for hypertension have highlighted difficulties in the disease’s architecture, management issues, and the need for transformation [15,16,17,18,19]. Over the last half-century, the treatment technique has remained virtually unchanged, and personalization of treatment has not gone beyond taking African ancestry and serum renin levels into account [20].

Furthermore, substantial genetic, molecular, and physiological research discoveries are not being integrated into screening, diagnostic, and management regimens. More than half of patients require numerous clinic visits at varied intervals to try dose titration, switching, or adding medicines until a satisfactory outcome is obtained, intolerable side effects develop, or no further progress appears likely [20]. Despite the high prevalence of hypertension, good health management must be devolved to the patients or machine learning-based intelligent systems [21].

In our research, we leverage the power of reinforcement learning and abundant electronic health records to dynamically recommend treatment prescriptions, which are personalized based on patient characteristics, including age, sex, body mass index, blood pressure, laboratory tests, and duration of hypertension patients with T2DM. At the initial state of the disease, doctors usually prescribe one medication for the initial treatment for hypertension [22]. Prescription can move to dual or triple therapy when the patient’s condition is not appropriate for mono or dual therapy, respectively [23]. Our proposed reinforcement learning based treatment recommendation system decides whether to use mono, dual, or triple therapy according to the state of the hypertension patients. We evaluated the performance of our personalized treatment recommendation model by lowering the occurrence of hypertension-related complications and blood pressure levels of patients who followed our model’s recommendation. Moreover, we compared our treatment recommendation with real-life doctors’ prescriptions to validate the reasonability of the recommendation.

## 2. Materials and Methods

### 2.1. Data Descriptions

Medical data for this research were provided by the National Health Insurance Sharing Service (NHISS) of Korea. The NHISS is a national agency providing the access of utilizing national health information data. The NHISS collects data under relevant guidelines and regulations, including obtaining informed consent from all participants (if participants are under 18, consent is obtained from a parent and/or legal guardian). The period of database is from 2003 to 2013. 

Currently the NHISS maintains and stores national records for healthcare utilization, prescriptions, and medical check-up. Medical check-up database contains major results from medical check-up and behavior and habitual data from questionnaire. Specifically, it includes the following contents: height, weight, waist, systolic and diastolic blood pressure, fasting plasma glucose, total cholesterol, triglyceride, HDL and LDL cholesterol, history (patient him/herself and family) of stroke, heart disease, hypertension, and diabetes, smoke status, drink habit, and exercise frequency.

We chose patients’ records from a national cohort data available in the NHISS database, and then filtered the hypertension patients with T2DM using the following criteria:

1. Diagnosis of hypertension according to the ICD-10 codes: I10;
2. Diagnosis of T2DM according to the ICD-10 codes: E10–E14;
3. Prescribed antihypertensive medications for more than 30 days;
4. Patients with complete medical check-up data upon appearance up to the end of data period or death, which includes total cholesterol (TC), body mass index (BMI), fasting plasma glucose (FPG), blood pressure (BP), smoke status, family history of hypertension and T2DM.

After processing, the total number of hypertension patients with T2DM was 14,934. From 1 January 2003, through to the date of their death or 31 December 2013, whichever came first, all participants were tracked.

Table 1 shows the statistics of the 14,934 hypertension patients with T2DM used. Male and female patients accounted for 56 percent and 44 percent of the data, respectively, with mean ages of 57 and 63 years. The period of having hypertension in female patients was 0.7 years longer than in male patients. BMIs were similar in both sexes; FPG levels exceeded 140 mg/dL, and TC levels were within normal limits. Both male and female patients had an average BP level of hypertension stage 1. Sixty-four percent and 41 percent of male and female patients are currently smoking, respectively. Lastly, 34 percent and 38 percent of male and female patients have a family history of hypertension, respectively.

### 2.2. Q-Learning

This study uses Q-learning, a data-driven model-free reinforcement learning approach to recommend medication treatment for hypertension patients with T2DM based on their current medical check-up measurements such as BP, FPG, BMI and smoke state.

Q-learning is adequate for determining the best action in a situation where neither the transition function nor the probability distribution of state variables is known. Q-learning is based on the estimation of a set of Q-values, which serves as a value function. Q-values are estimated for each state–action $\left(s_{t},a_{t}\right)$ combination in the Q-learning method [24]. The status of the environment (hypertension patient state, $s_{t}$) should be known in order to choose the optimal action (antihypertensive medication, $a_{t}$) when the final Q-values are calculated. Q-values are set to an arbitrary real number at the start of the process. The reinforcement learning agent then calculates a reward value for each state and action combination at iteration t. Equation (1) shows the essence of the algorithm, which is the iterative process of updating Q-values as a function of the immediate reward $r_{t}$ and Q-values of the next state-action pair $Q\left(s_{t+1},a_{t+1}\right)$. γ is the discount factor that regulates the effect of future rewards relative to current rewards with range from 0 to 1.

(1) $$Q\left(s_{t},a_{t}\right)\leftarrow r_{t}+\gamma \;\underset{a_{t+1}}{\max}\left\{Q\left(s_{t+1},a_{t+1}\right)\right\}$$

The Q-values are updated so that the series of Q-values converges to an optimal action-value function Q*, irrespective of any policy [25]. One of the most appealing features of Q-learning is its flexible sampling strategies for generating state–action pairs. One of the common sampling methods is the ε-greedy action selection, defined in Equation (2).

(2) $$a_{t}=\{\begin{array}{c} arg\;\underset{a\in A}{\max}\;Q\left(s_{t},a\right)\\ a\;\sim \;A \end{array}\;\begin{array}{c} with\;probability\;1-\varepsilon ,\\ otherwise. \end{array}$$

where ε∈(0,1]. The ε-greedy policy makes an agent select either the greedy action with the probability of 1−ε, or otherwise it chooses a random action from the action space. The randomness ensures that the agent experiments with alternative activities from time to time, resulting in a greater return in the end.

#### 2.2.1. State S

According to the World Health Organization report, several factors such as diet, alcohol usage, physical activity, BMI, age, and smoking status can affect the blood pressure level of hypertension patients [26].

In this research, we chose 5 components that affect the hypertension patients with T2DM shown in Equation (3).

(3) $$S^{t}=(S_{COMPLICATIONS}^{t},S_{AGE}^{t},S_{PERIOD}^{t},S_{BP}^{t},S_{BMI}^{t});\;t=1,\ldots T$$

where $S_{COMPLICATIONS}^{t}$ is a state of hypertension-related complications at time t for t=1,…T. If a patient has one or more complication(s) at time t, it is 1; otherwise, it is 0. We considered complications including heart and chronic kidney diseases, as shown in Table 2. We also defined the International Classification of Diseases (ICD-10) codes of complications.

$S_{AGE}^{t}$ is the age of the patients at time t for t=1,…T. If a patient’s age is less than 55 years old, at time t, it is 1; otherwise, it is 0. We considered the boundary age as 55 years because the risk of diabetes increases for patients aged > 55 years [27].

$S_{Period}^{t}$ represents the time elapsed since the onset of diabetes. If the time is less than or equal to 4 years, it is 0. If the time ranges between 5 and 8 years, it is 1; otherwise, it is 2.

$S_{BP}^{t}$ represents the BP level. We divided the BP level into three levels according to the BP measurement, as shown in Table 3. If the level is prehypertension, it is 0; stage 1, 1; and stage 2, 2.

$S_{BMI}^{t}$ represents the BMI. If the level is below 18.5, it is 0; between 18.5 and 25, it is 1; and above 25, it is 2. These three levels were divided into the following stages: underweight, normal, and overweight.

The total number of states in this model was 108 (2 × 2 × 3 × 3 × 3). We were considering the factors that affect hypertension that we need to consider for recommending treatment options.

#### 2.2.2. Action A

**Figure 1:**
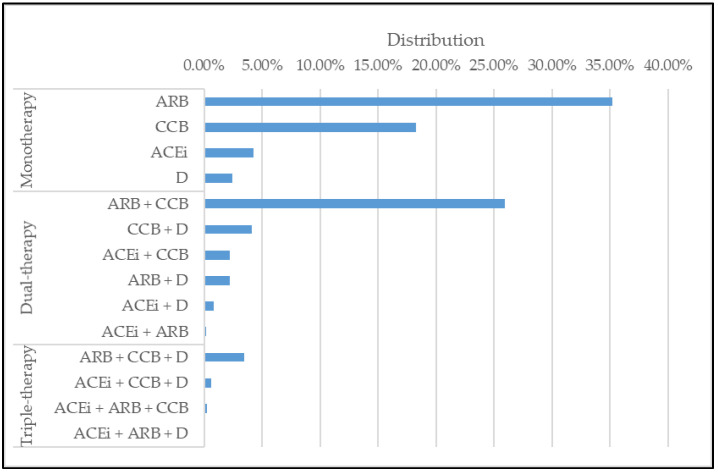
Distribution of actions in database.

The action of our model comprised prescriptions for hypertension patients with T2DM. There are 4 classes of antihypertensive medications, which include, Diuretics (D), ACE inhibitors (ACEi), Angiotensin II receptor blockers (ARB), and Calcium channel blockers (CCB) [28]. We selected 14 medications that consisted of mono, dual, and triple therapies made by 4 medications as shown in Table 4. We also observed the frequency of each action used in the database in Figure 1.

#### 2.2.3. Reward R

We adopted quality-adjusted life-year (QALY) in our model as a reward to improve patients’ expected time in a healthy state. QALY is a disease load metric that considers both the quality and quantity of life lived. It is used to measure the value of medical therapies in economic evaluation. One QALY is the equivalent of a year of excellent health. The QALY score ranges from 1 (excellent health) to 0 (dead). QALY can be used to guide health insurance coverage decisions, treatment decisions, program evaluations, and future program priorities [29].

The reward function $R\left(a,s^{\prime }\right)$ of our proposed model is shown in Equation (4), where a is an action and $s^{\prime }$ is the resulting state.

(4) $$R\left(a,s^{\prime }\right)=R^{WTP}[\left(1-d^{COMPLICATIONS}\left(s^{\prime }\right)\right)\left(1-d^{AGE}\left(s^{\prime }\right)\right)\left(1-d^{PERIOD}\left(s^{\prime }\right)\right)$$

$$\left(1-d^{BP}\left(s^{\prime }\right)\right)\left(1-d^{BMI}\left(s^{\prime }\right)\right)]-C^{MED}$$

$R^{WTP}$ is the value of willingness to pay for a QALY of 1. We considered the following five decrement factors: $d^{COMPLICATIONS}\left(s^{\prime }\right)$, $d^{AGE}\left(s^{\prime }\right)$, $d^{PERIOD}\left(s^{\prime }\right)$, $d^{BP}\left(s^{\prime }\right)$, and $d^{BMI}\left(s^{\prime }\right)$ due to complications, age, period, BP, and BMI levels. The details are shown in Table 5. Lastly, $C^{MED}\left(a\right)$ represents the cost of medication used [30].

The decrement values are referenced from other studies [31,32,33,34].

## 3. Hypertension Treatment Recommendation Results

### 3.1. Hypertension Treatment Recommendation Results

**Figure 2:**
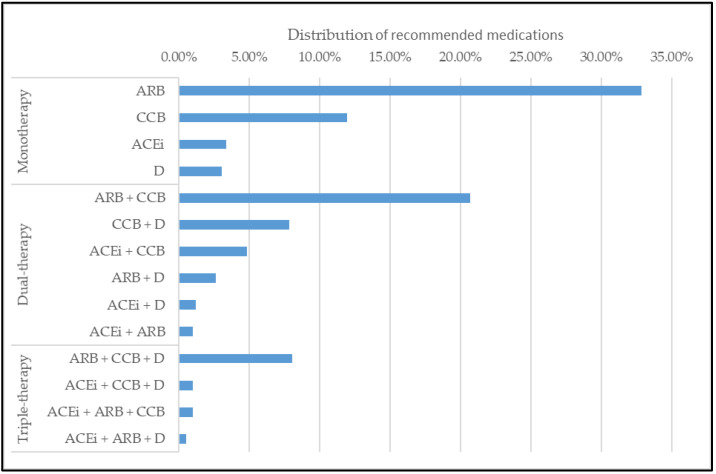
Distributions of recommended medications by model.

In this section, we observe the results of medications recommended by our model. The model’s recommendation is similar with the prescriptions from a database that is made by doctors. However, our model recommended more dual and triple therapies than doctors. The distribution of recommended medications is shown in Figure 2.

**Figure 3:**
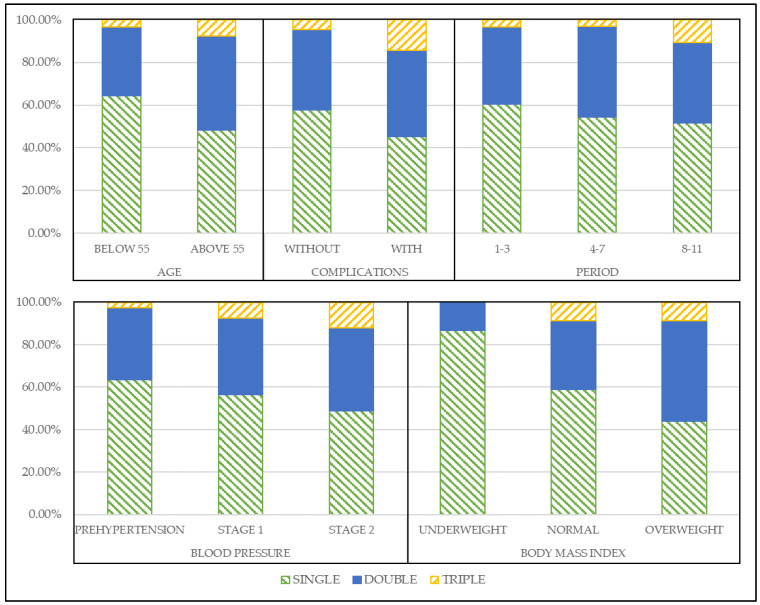
The trend of medication recommendations by each state component.

We also arranged the medications in mono, dual, and triple therapies to observe the medications’ shift trend and verify the usage of multiple medications. The recommended actions for each state component are shown in Figure 3.

In Figure 3, we can observe the trend of the recommended medications that shift from monotherapy to dual and triple therapies as the patients’ condition worsens. In the age state, when the age was above 55 years old, monotherapy decreased by 16%, while the portion of dual and triple therapy increased by 12% and 4%, respectively. The patients with complications were also recommended more dual and triple therapies by 3 and 10% than without complication, respectively. In the period state, the recommendation trends are similar with other states, but we observed that in 8 to 11 years, triple therapy recommendation increased by 7% compared to other periods. Most importantly, in blood pressure state, as the level of blood pressure becomes higher, more dual and triple therapies are recommended. Lastly, it is the same with BMI state, overweight patients receive more dual and triple therapy recommendations than underweight and normal state patients by most 24% and 8%, respectively.

We excluded the recommendation results of female patients, since they have a similar trend with the male patient, to improve readability (results are available upon request).

With this observation, we verified why our model’s recommendations have more dual and triple therapy than prescriptions from the database. Furthermore, we validated our model’s recommendations in the next section to prove the performance of our results.

### 3.2. Concordance Rate Validation

This section validates the result by the concordance rate between the model’s recommendation and the doctor’s prescription. To rate the score, we checked if the model’s recommendation and doctor’s prescription exactly match each state. For the doctor’s prescriptions, the most frequent prescriptions for each state were compared with the recommendation of our model. Among 108 states, we counted the number of matched states and calculated the percentage. The results shown in Table 6.

The results show a concordance rate of 85.18% and 81.48% for male and female patients, respectively. As prescription may vary depending on the patient’s condition and doctor’s preference, we believe that our model’s recommendation is reasonable for hypertension treatment.

### 3.3. Medication Possession Ratio Validation

In this section, we obtained the medication possession ratio (MPR) of our model’s recommended medications to patients to validate the compliance and adherence. Medication adherence is defined as the extent to which a patient takes the medication prescribed recommended by the provider [35]. In the previous studies, MPR-related adherence measures were generally defined as the proportion of a time period where a medication supply is available [36,37,38]. MPR can be calculated using Equation (5) [35].

(5) $$\mathrm{Medication}\;\mathrm{posseession}\;\mathrm{ratio}\;\left(\mathrm{MPR}\right)=\frac{\mathrm{Total}\;\mathrm{days}\;\mathrm{supply}\;\mathrm{of}\;\mathrm{medication}}{\mathrm{Number}\;\mathrm{of}\;\mathrm{days}\;\mathrm{in}\;\mathrm{period}}\times 100$$

In our research, we used the MPR to observe the adherence of patients to our model’s recommended medications to validate that the patients really followed our recommended actions to improve their health condition. The result of MPR is shown in Table 7 and Table 8 for male and female patients, respectively.

The result shows that for male patients, the mean MPR for all numbers of medication have exceeded 61%. Same with female patients, the mean MPR of every number of medications exceeded 66%. With these results, we showed that our recommended medication complied with patients.

### 3.4. Model Concordance Rate vs. Hypertension-Related Complication Occurrence

**Figure 4:**
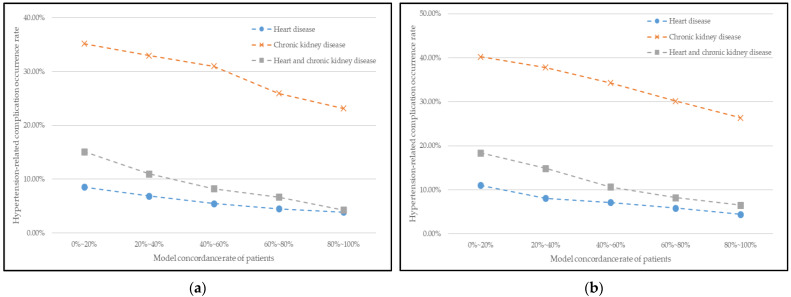
Relationship between patients’ model concordance rate and complication occurrence for (**a**) male patients and (**b**) female patients.

In this section, we validate the performance of our recommended medication by observing hypertension-related complications’ occurrence. Figure 4 illustrates the relationship between the patient’s model-concordant rate and hypertension-related complications occurrence rate for male and female patients. The patients were separated into different groups by 20 percent based on their model-concordant rate, and the average occurrence rate of complications in each group was calculated.

In Figure 4, the curves reflect a declining trend in general. In other words, the model-concordant rate and the occurrence rate of complications have a negative relationship; the greater the patient’s model-concordant rate, the lower the occurrence rate of complications. Therefore, our model’s recommendation positively affects hypertension treatment and also the management of good health conditions.

### 3.5. Model Concordance Rate vs. Blood Pressure Level

**Figure 5:**
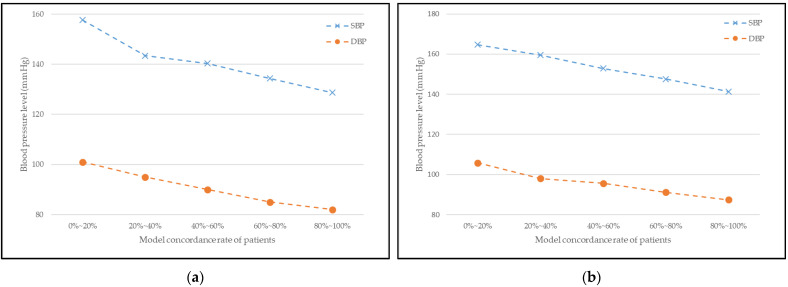
Relationship between patients’ model concordance rate and blood pressure level for (**a**) male patients and (**b**) female patients.

In this section, we validate the performance of our recommended medication by observing the variation in the patients’ blood pressure level. Figure 5 illustrates the relationship between the patient’s model-concordant rate and blood pressure levels for male and female patients. As in Section 3.1, the patients were separated into different groups by 20 percent based on their model-concordant rate, and this time, the average blood pressure level in each group was calculated.

In Figure 5, the curves generally reflect a decreasing trend. This means that the model-concordant rate and the blood pressure levels are inversely proportional; the greater the patient’s model-concordant rate is, the lower the blood pressure level is. Therefore, our model’s recommendation positively affects hypertension treatment and also the management of proper blood pressure level.

### 3.6. Performance Comparison with Other Reinforcement Learning Model

**Figure 6:**
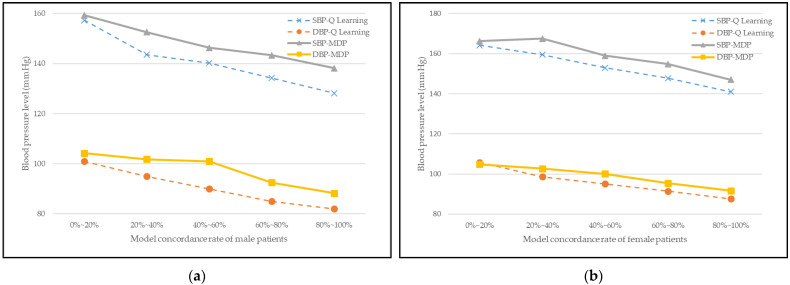
Relationship between patients’ model concordance rate and blood pressure level of Q-learning and MDP for (**a**) male patients and (**b**) female patients.

We compared our proposed model with another reinforcement algorithm that is popular and previously studied by us, namely, the Markov decision process (MDP) [39]. The performance comparison metrics used are the concordance rate with doctor’s prescription and blood pressure level variation. The results are shown in Table 9 and Figure 6.

Table 9 shows the concordance rate result between the models’ recommendation and the doctor’s prescription. We checked if the model’s recommendation and doctor’s prescription exactly match each state to rate the score.

The results show a concordance rate of 85.18% and 81.48% for our proposed Q-learning model for male and female patients, respectively. The MDP model results are 78.7 and 75.93% for male and female patients, respectively. We verified that our model has good performance by over-performing in the concordance rate compared to another reinforcement learning algorithm.

Figure 6 shows the variation of patients’ blood pressure level according to concordance rate of Q-learning and MDP recommendation. The patients were separated into different groups by 20 percent based on their model-concordant rate, and this time, the average blood pressure level in each group was calculated. In Figure 6, the curves generally reflect a decreasing trend for both models. However, the decreasing gap of our proposed Q-learning is larger compared to the MDP model for both male and female patients. Therefore, our proposed reinforcement learning model’s recommendation has greater effects on hypertension treatment.

## 4. Discussion

We proposed a reinforcement learning-based hypertension treatment recommendation model with South Korean medical records. We choose Q learning as our algorithm for the recommendation because it is the fundamental reinforcement learning model. With the proposed model, we recommend antihypertensive medications, whether to choose mono, dual, or triple therapy according to the state of the patients. Using the Korean medical records, we observed that the model’s recommended actions change from monotherapy to dual and triple therapies as patients’ condition worsens. For example, in age state, as the age above 55 years old, monotherapy recommendation was decreased while dual and triple therapy increased. Hypertension-related complications also affect the recommendation trend by having more dual and triple therapies. In the period state, as patients have a longer period of hypertension, we observed that triple therapy recommendations increased. For blood pressure state, as the level of blood pressure goes higher, dual and triple therapy recommendations are getting higher. Lastly, overweight patients are recommended for more dual and triple therapy recommendations than underweight and normal state patients in BMI state.

Our recommended actions are validated by computing the concordance rate between the model’s recommendation and the doctor’s prescription. This rate is calculated by comparing the model’s recommendation and doctor’s prescription if they match each other. The reason for checking this rate is to verify that our model recommends appropriate medications for accorded states. The results showed that the concordance rate was higher than 80% for all patients. We could claim that the recommendation is reasonable considering that prescription may vary depending on the patient’s condition and doctor’s preference. We also worked on MPR validation to verify patients’ compliance and adherence to our model’s recommended medications for further verification. Patients’ adherence to medication is also related to the excellent maintenance of hypertension treatment. If the MPR is in an acceptable range, we can claim that patients have complied with our recommended medications. As shown in the results section, the mean MPR for mono, dual, and triple therapy medication exceeded 61% for male patients. As well as female patients, the mean MPR of all numbers of medicine exceeded 66%. With these results, we showed that our recommended medication complied with patients.

After proving our model’s recommendations are reasonable and complied with patients, we validate the hypertension maintenance performance by observing the relationship between the patient’s model-concordant rate and hypertension-related complications occurrence and variation of blood pressure level. The results showed that the relationship curves reflect a declining trend in general. In other words, the model-concordant rate and the occurrence rate of complications and blood pressure levels have a negative relationship; the greater the patient’s model-concordant rate, the lower the occurrence rate of complications and blood pressure levels. Therefore, our model’s recommendation positively affects hypertension treatment and also the management of good health conditions.

Lastly, we compare our model’s performance with another reinforcement learning which is MDP. The performance comparison metrics used are concordance rate with doctor’s prescription and blood pressure level variation. The results showed that our proposed model has a higher concordance rate than MDP by 7% and 4% for male and female patients, respectively. Moreover, for the variation of patients’ blood pressure level according to the concordance rate of Q learning and MDP recommendation, we observed that as the rate of concordance increases, the blood pressure level decreases for both models. However, the decreasing gap of our proposed Q learning is larger compared to the MDP model for both male and female patients. Therefore, we verified our proposed hypertension treatment recommendation model using reinforcement learning has high-quality performance.

The limitation of our study is that we only deal with one kind of medical record, which our national health institute provides. It could also be the strength of our paper that only a few studies have utilized the health records of South Koreans for medical machine learning research. However, it is better to acquire at least two health databases to verify results in various methods. Moreover, we could not use hemoglobin A1c level, one of the important factors for diabetes patients, due to the absence in the database. We decided to use BMI levels to represent the patient’s condition since it is the risk factor for diabetes and hypertension.

In future work, we would like to acquire other EHRs by collaborating with hospitals in Korea or databases from other countries to verify our proposed model and compare the result by race. Furthermore, by having various databases, we can broaden the disease area to a large point of view. In this research, we only cover hypertension patients with diabetes, but we plan to expand to general hypertension or other severe diseases in future studies.

Finally, it is crucial to deal with the uncertainty of the action or prescription in the clinical practice. Our model studies and learns from the many rounds of the database to get closer to the realistic and precise prescription, leading to optimal actions. Therefore, we believe that doctors could apply data-based machine learning results to their research field to assist in clinical practice.

## 5. Conclusions

This research suggested a reinforcement learning-based antihypertensive medication recommendation system for hypertension patients with T2DM. This research aims to address the challenge of precision medicine utilizing enormous electronic health data and machine learning, which led to the introduction of a reinforcement learning model called Q-learning. We constructed the model to be as realistic as possible by including the risk factors of hypertension as a state and a combination of antihypertensive medication as action. We used the 11-year electronic health records of a South Korean database with 1 million patients per year to create the model. We delicately designed the states, actions, and reward functions to simulate our proposed model.

Our results highlight that the hypertension treatment recommended by our Q-learning model is significant, as it correctly predicts the trend of a shift from monotherapy to dual and triple therapy as the patient’s condition worsens, because even in the real world, when a patient’s condition does not progress, doctors increase the number of medications and prescribe them in combination. We also proved that the performance of our proposed model by lowering blood pressure level of patients.

Based on our findings, making the appropriate decision about the correct number and type of antihypertensive medicine could help postpone or prevent hypertension-related complications such as heart disease, chronic renal disease, and both. Furthermore, our reinforcement learning approach can help minimize patient stress, lower healthcare costs, and improve the overall quality of life by reducing the time it takes to obtain a successful hypertension treatment. To conclude, we believe that our proposed hypertension treatment recommendation model could assist doctors in prescribing appropriate antihypertensive medications.

## Figures and Tables

**Table 1:** Statistics of data set used.

Category	Male	Female
Sex (%)	56	44
Age, mean (SD)	57 (22)	63 (23)
Period of having hypertension (years), mean (SD)	7.1 (3.4)	7.8 (2.8)
BMI (kg/m^2^), mean (SD)	27.9 (2.5)	28.2 (3.2)
FPG (mg/dL), mean (SD)	143.8 (53.2)	147.6 (51.6)
TC (mg/dL), mean (SD)	184.2 (47.3)	191.2 (47.9)
Systolic BP (mmHg), mean (SD)	132.5 (25.8)	138.5 (26.7)
Diastolic BP (mmHg), mean (SD)	84.8 (17.6)	87.9 (16.8)
Smoker (%)	64	41
Family history of hypertension (%)	34	38

**Table 2:** Types and ICD-10 codes of hypertension complications.

Types of Complications	ICD-10 Codes
Heart disease	I11
Chronic kidney disease	I12
Heart and chronic kidney disease	I13

**Table 3:** Blood pressure level category.

Blood Pressure Category	Systolic (mmHg)	Diastolic (mmHg)
Prehypertension	120–139	80–89
Stage 1	140–159	90–99
Stage 2	160 or higher	100 higher

**Table 4:** Action descriptions.

Type	No.	Medication
Monotherapy	1	ARB
	2	CCB
	3	ACEi
	4	D
Dual therapy	5	ARB + CCB
	6	CCB + D
	7	ACEi + CCB
	8	ARB + D
	9	ACEi + D
	10	ACEi + ARB
Triple-therapy	11	ARB + CCB + D
	12	ACEi + CCB + D
	13	ACEi + ARB + CCB
	14	ACEi + ARB + D

**Table 5:** Reward value descriptions.

Notations	Descriptions	Decrement Values
$d^{COMPLICATIONS}\left(s^{\prime }\right)$	Utility decrement value associated to complications	0:0 1:0.248
$d^{AGE}\left(s^{\prime }\right)$	Utility decrement value associated to age	[0, 55):0.08 [55, ~):0.129
$d^{PERIOD}\left(s^{\prime }\right)$	Utility decrement value associated to hypertension period	[1, 4):0.078 [4, 8):0.085 [8, 11):0.112
$d^{BP}\left(s^{\prime }\right)$	Utility decrement value associated to blood pressure	Prehypertension: 0.034 Stage 1:0.125 Stage 2:0.278
$d^{BMI}\left(s^{\prime }\right)$	Utility decrement value associated to BMI	[0 ,18.5):0.028 [18.5, 25):0.07 [25, ~):0.172
$C^{MED}\left(a\right)$	Cost of medication	It varies depends on actions

**Table 6:** Concordance rate between model’s recommendation and doctor’s prescriptions.

Gender	No. of Matched States	Concordance Rate
Male	92	85.18%
Female	88	81.48%

**Table 7:** MPR of male patients.

	Mono Therapy	Dual Therapy	Triple Therapy
Min	34%	27%	25%
Max	85%	76%	78%
Mean	67%	61%	63%

**Table 8:** MPR of female patients.

	Mono Therapy	Dual Therapy	Triple Therapy
Min	37%	31%	29%
Max	88%	79%	81%
Mean	71%	68%	66%

**Table 9:** Concordance rate of Q-learning and MDP between model’s recommendation and doctor’s prescriptions.

Gender	Q-Learning	MDP
Male	85.18%	78.7%
Female	81.48%	75.93%

## Data Availability

The datasets generated during and/or analyzed during the current study are not publicly available due to containing patient information collected by National Health Insurance Sharing Service, which requires payment for access. However, sample data are available from the corresponding author on reasonable request.
